# The associations of perceived neighborhood disorder and physical activity with obesity among African American adolescents

**DOI:** 10.1186/1471-2458-13-440

**Published:** 2013-05-04

**Authors:** Akilah Dulin-Keita, Herpreet Kaur Thind, Olivia Affuso, Monica L Baskin

**Affiliations:** 1 Institute for Community Health Promotion, Department of Behavioral and Social Sciences, Brown University, Providence, RI, USA; 2 Centers for Behavioral and Preventive Medicine, Department of Psychiatry & Human Behavior, Brown Alpert Medical School and the Miriam Hospital, Providence, RI, USA; 3 Department of Epidemiology, University of Alabama at Birmingham, Birmingham, AL, USA; 4 Division of Preventive Medicine, University of Alabama at Birmingham, Birmingham, AL, USA

**Keywords:** Neighborhood, Physical activity, Obesity, Adolescent, Race/ethnicity

## Abstract

**Background:**

According to recent research studies, the built and socioeconomic contexts of neighborhoods are associated with African American adolescents’ participation in physical activity and obesity status. However, few research efforts have been devoted to understand how African American adolescents’ perceptions of their neighborhood environments may affect physical activity behaviors and obesity status. The objective of the current study was to use a perceived neighborhood disorder conceptual framework to examine whether physical activity mediated the relationship between perceived neighborhood disorder and obesity status among African American adolescents.

**Methods:**

The data were obtained from a cross-sectional study that examined social and cultural barriers and facilitators of physical activity among African American adolescents. The study included a sample of 101 African American adolescents age 12 to 16 years and their parents who were recruited from the Birmingham, Alabama metropolitan area. The primary outcome measure was obesity status which was classified using the International Obesity Task Force cut off points. Moderate-to-vigorous physical activity was assessed via accelerometry. Perceived neighborhood disorder was assessed using the Perceived Neighborhood Disorder Scale. Mediation models were used to examine whether the relationship between neighborhood disorder and obesity status was mediated by physical activity.

**Results:**

Perceived neighborhood disorder was significantly and positively related to obesity status and moderate-to-vigorous physical activity was inversely associated with obesity status. However, there was no evidence to support a significant mediating effect of moderate-to-vigorous physical activity on the relationship between neighborhood disorder and obesity status.

**Conclusion:**

Future studies should longitudinally assess perceived neighborhood disorder characteristics and childhood adiposity to examine the timing, extent, and the mechanisms by which perceived neighborhood disorder characteristics increase the risk of obesity.

## Background

Participation in physical activity is a key health promoting behavior that prevents weight gain and reduces the risk for obesity [1,2]. However, there are age-related declines in physical activity levels among adolescents with peak declines evidenced between 15 to 18 years of age [3-6]. While both national and international guidelines suggest that adolescents engage in a minimum of 60 minutes of moderate-to-vigorous physical activity daily, less than 10 percent of all US adolescents age 12 to 15 years meet this recommendation [2,5,7]. When the physical activity estimates are examined across racial/ethnic groups, the research findings suggest that African American adolescents are unlikely to meet the recommendations for moderate-to-vigorous physical activity [7] and African American adolescents in this age group are more likely to be overweight or obese relative to other racial/ethnic groups [5]. Data from the National Health and Nutrition Examination Survey (NHANES 2009–2010) indicate that 41.2 percent of African American adolescents age 12 to 19 years are either overweight or obese (23.7 percent are obese) relative to 30.0 percent of white adolescents age 12 to 19 years (16.1 percent are obese) [8]. These research findings indicate that African American adolescents’ declines in physical activity levels are consistent with national declines among US adolescents, but also indicate that African American adolescents encounter higher levels of obesity.

Features of the neighborhood environment may contribute to the declines in physical activity and the higher levels of obesity among African Americans. Relative to other racial/ethnic groups, African American adolescents are more likely to reside in low-income urban areas that have more built environment barriers such as poor housing stock, sidewalks, street design and greater traffic density within neighborhoods [9,10]. African American adolescents also live in neighborhoods with higher levels of social, physical and economic disorder which include features such as lack of trust among neighbors, abandoned buildings, crime, graffiti and concentrated poverty that contribute to social instability [10-13]. While research findings indicate that built environment features associated with physical activity such as less availability of parks, poor neighborhood walkability and street connectivity are associated with low levels of physical activity [14-17], the associations of the built environment with obesity are less clear. Support for the association of the built environment with adolescent obesity is inconsistent with a significant association found among some, [1,18-20] but not all research [21,22]. Additional research that examines the social contexts of the neighborhood suggests that low socioeconomic status may contribute to low levels of physical activity [23,24]. However, the associations of neighborhood socioeconomic status with obesity are inconclusive; some studies indicate that neighborhood level socioeconomic status is inversely associated with obesity [24-28], whereas others do not find significant associations [29,30].

While the literature on the roles of the neighborhood built and economic environments provide compelling evidence to develop further insights into neighborhood level effects on physical activity and obesity, the subjective contexts of African American adolescents’ neighborhood perceptions and the relationships with physical activity participation and obesity status, are less researched. Although social factors such as neighborhood safety, social cohesion, and perceptions of crime have been examined among multi-ethnic cohorts of adolescents participating in research studies such as the Healthy Passages Study [31], additional neighborhood features that might specifically affect African American adolescents’ participation in physical activity and obesity outcomes warrant attention.

The purpose of the current study was to examine the relationships of perceived neighborhood physical and social disorder with physical activity and obesity status among African American adolescents. To examine this objective, we utilized the conceptual framework developed by Burdette and Hill [32], which suggests that perceived neighborhood disorder is indirectly related to obesity through a series of mediating factors. According to the model outlined by Burdette and Hill [32], observable indicators of neighborhood social and physical disorder such as crime, public loitering, vandalism, graffiti, and abandoned buildings, are associated with psychological and physiological distress. These indicators of neighborhood disorder may induce psychological distress because of heightened perceptions of potential risks to physical safety and/or victimization. Individuals who experience psychological distress may not engage in regular physical activity and are potentially more likely to engage in obesogenic sedentary behaviors and have poorer diet quality (e.g. greater fat and sugar intakes). Additionally, the increase in psychological distress may result in chronic activation of the physiological stress response and may lead to negative coping such as poorer diet quality and less participation in physical activity. While the conceptual framework outlines psychological and physiological distress as pathways to obesity, the current study did not include measures of physiological or psychological distress. However, the perceived neighborhood disorder conceptual framework is applicable to the current study aims as there are additional pathways through which neighborhood disorder may contribute to obesity. The research findings of Evenson et al. [33] suggest that disordered neighborhood environments may directly discourage physical activity within neighborhoods due to fear of crime and/or the absence of physical activity promoting resources. Therefore we hypothesized that among African American adolescents, the association between perceived neighborhood disorder and obesity would be mediated by physical activity.

## Methods

Data for the current study are from a cross-sectional study that examined the social and cultural factors that influence African American adolescents’ participation in physical activity.

### Subjects

To meet eligibility criteria for the study, adolescents had to be 12 to 16 years of age, self-identify as African American and present with no physical or mental impairment. Adolescents and their parents were recruited from the Birmingham, Alabama metropolitan area, where the population is predominantly African American (73.4 percent) and where 32.7 percent of families with children have incomes below the poverty level (US Census Bureau: American FactFinder. Available at http://factfinder2.census.gov/faces/nav/jsf/pages/community_facts.xhtml). To recruit study participants, we used passive recruitment methods, where participants identified themselves as potential participants, and active recruitment methods, where our research team identified and targeted potential participants [34]. The passive recruitment strategies included posted flyers (with the contact information of the project coordinator provided on the flyer) at local recreational centers, churches, community centers, newspaper advertisements posted on the University website, and word-of-mouth advertising by previous study participants. Active recruitment for this study included staff initiated phone contacts with adolescents who participated in the phase 1 qualitative portion of this study and targeted recruitment of adolescents from one afterschool program at a community center. For this study, passive recruitment via word-of-mouth was the most effective recruitment strategy. All study materials, methods, and study ethics were approved by the Institutional Review Board of the University of Alabama at Birmingham.

To identify an acceptable sample size for this study, we generated a series of multivariate linear regression models using the PASS statistical software. The sample size was calculated based on a significance level α = 0.05 with varying levels for beta such that Power (1- Beta) equals 0.90, 0.85, or 0.80. The sample size calculations were adjusted for the multivariate nature of the analyses by including a conservative estimate of the R^2^ (R^2^ = 0.10) that is attained when family income, a primary independent variable used in our proposed sampling procedure, is regressed on 10 other independent variables in the regression models. The sample size calculations are strong evidence that the proposed sample size of 120 provided enough power to conduct the multivariate analysis for this study.

Data were collected in Spring 2011 and all study related events were held at the University of Alabama at Birmingham, Division of Preventive Medicine. Of the 145 adolescents screened to be eligible, 116 (54 percent female and 46 percent male) completed the study. The remaining 29 eligible participants reported scheduling conflicts as the main reason for not participating in the study meetings.

### Procedures

Parents and adolescents who expressed interest in the study were invited to participate in a 90-minute study meeting. Prior to study participation, trained staff obtained informed consent from the parents and assent from the adolescents. During the meeting, adolescents and their parents completed self-administered paper surveys and the adolescents received accelerometers, which they returned approximately one week after the initial meeting. Parents and adolescents received $10 each for completing the surveys and adolescents received an additional $25 dollars for wearing and returning the accelerometer.

### Measurements

#### Dependent variables

##### Body mass index (BMI)

Trained research staff collected anthropometric measurements. To obtain height measurements, adolescents’ were measured without shoes using a portable stadiometer (Seca 213). Height was measured to the nearest 0.1 cm. To obtain weight measurements, adolescents wore light clothing and were weighed without shoes to the nearest 0.1 kg using a digital scale (Seca 813). Two independent measurements were taken for weight and the average of the two was used. BMI was calculated using the formula kg/m^2^.

##### Obesity status

BMI was collapsed into categories of normal weight, overweight or obese using the International Obesity Taskforce cut offs which average data from six countries (Brazil, Great Britain, Hong Kong, Netherlands, Singapore and the United States) [35]. The age-sex specific centile curves correspond to adult cut off points of 25 kg/m^2^ (overweight) and 30 kg/m^2^ (obese) at age 18 years of age.

#### Independent variables

##### Perceived neighborhood disorder

Parents and adolescents self-reported neighborhood conditions using the full 15-item version of the Ross and Mirowsky Neighborhood Disorder Scale [36]. Because there were no significant differences in mean neighborhood disorder scores between parents and adolescents, we used adolescent responses for all analyses. The Neighborhood Disorder statements assessed perceptions of physical and social disorder and a prosocial neighborhood environment. Examples of the scale items include ‘there are too many people hanging around on the streets near my home,’ ‘my neighborhood is noisy,’ and ‘I can trust most people in my neighborhood.’ A Likert format (‘strongly agree = 4 to ‘strongly disagree’ = 1) was used to collect response options for the neighborhood disorder scale. For this scale, the prosocial neighborhood environment items were reverse coded. The responses for the items were summed and the possible score values were 15 to 60, with higher scores indicating greater perceived neighborhood disorder. The Cronbach’s alpha was α = 0.913. We constructed quartiles to examine outcomes of interest by quartiles of perceived neighborhood disorder.

### Physical activity

#### Objective assessment

We assessed adolescents’ physical activity via accelerometry. Adolescents wore the Actigraph uniaxial accelerometers (Model GT1M; Actigraph Manufacturing Technology Inc., Pensacola, FL, USA) which have demonstrated high reliability for this population [37]. The research staff explained the purpose of the accelerometer and demonstrated its use at the study meeting. Adolescents were asked to wear the accelerometers at their waist for 7 consecutive days including nights and were instructed to remove them only for bathing or swimming purposes. To improve compliance with the accelerometer protocol, at least two reminders were sent through email, text message, or phone call based on the participants’ desired method of contact. We followed the protocols of Toriano et al. [7] for collecting accelerometer data. Epoch length was set at one minute and data were expressed as counts per minute (counts min^-1^). Accelerometry data were considered valid if counts were present for at least three days with at least 8 hours of recording per day. We used validated child and age specific algorithms to determine physical activity [7]. We converted the accelerometer data to Metabolic Equivalents (METs). Daily and total counts per minute were summed and averaged as minutes spent in sedentary (1 to 1.5 METs), light (1.5 to 4 METs), moderate (4 to 7 METs) and vigorous (> 7 METs) physical activity. We used these criteria for METs to account for the higher resting energy expenditure of adolescents [38]. Because children engaged in few minutes of vigorous physical activity, values were combined with moderate physical activity.

#### Subjective assessment

Adolescents recorded their daily physical activity for two weekdays and one weekend day using the validated 3-Day Physical Activity Recall log (PDPAR) [39,40]. Each log was divided into 30 minute blocks and the adolescents recorded their main activity for each block. Adolescents provided open-ended responses, which were used to identify types of physical activity that adolescents engaged in while wearing accelerometers. Trained staff coded the open-ended responses to the 71 activities included in the 3DPAR logs using established methodology [39]. The staff then calculated the average time spent on each activity.

#### Covariates

Age, sex, and socioeconomic status were examined as possible covariates. Parents reported for the child the age and sex of the child. Age was examined as a continuous measure and sex was examined as a categorical variable. To obtain measurements of socioeconomic status, parents self-reported their total annual household income and the highest level of education completed by any adult in the household (1 = less than high school, 2= complete high school, 3 = some college, and 4 = college graduate). For analyses, income and education were treated as ordinal variables.

### Data analysis

All variables were examined for normality using the Shapiro-Wilk test. Because there were some variables that were not normally distributed, we conducted nonparametric statistical tests where appropriate. Initially, we conducted analyses stratified by gender but found no significant differences; thus, all analyses presented include results for both girls and boys together. Means, standard deviations, and percentages were computed for the dependent and independent variables by quartiles of perceived neighborhood disorder. Simple bivariate correlations for all independent variables were evaluated to test for multicollinearity [41,42]. None of the variables were significantly correlated and all independent variables and covariates were included for analysis. One-way ANOVA, Kruskal-Wallis tests or Chi-square were conducted to assess significant differences for the dependent and independent variables α<.05, by quartiles of perceived neighborhood disorder.

We conducted single mediation analyses to examine whether the relationship between neighborhood disorder and body mass index was significantly mediated by physical activity. In these analyses, we assessed neighborhood disorder as a continuous variable and examined adolescent obesity status categories (1 = normal weight, 2 = overweight and 3 = obese) that corresponded to the International Obesity Taskforce centile curves. To examine the indirect (mediational) effect in these models, we used the bootstrap procedures outlined by Preacher and Hayes [43] and we used their publicly available Indirect SAS macros available at http://www.afhayes.com/spss-sas-and-mplus-macros-and-code.html. Bootstrapping is a nonparametric procedure that is robust to departures from the assumptions of normally distributed sampling distributions and is the most appropriate method for small sample sizes [43]. We included the bias-corrected (BC) and bias-corrected and accelerated (BCa) confidence intervals to account for the asymmetric distribution of point estimates [43]. We set the BCa confidence intervals at 0.95 with 5000 resamples for bootstrapping [43]. To model the quadratic association of education with obesity, we created a variable, squared-education for each participant and this non-linear effect was added as a covariate along with education in the mediation models. As recommended by Preacher and Hayes, all of the point estimates presented in the mediation analysis are the unstandardized beta coefficients [43]. The probability criterion was set at α <0.05. We used SAS version 9.2 (SAS Institute Inc, Cary, NC) for all analyses.

## Results

### Descriptives

Of the 116 adolescents who participated in the study, 15 were excluded due to missing/incomplete data (nine were missing information on perceived neighborhood disorder, four were missing information on income, one was missing information on BMI and one did not have accelerometer data for three days). There were no significant differences between adolescents with missing data and participants included in data analyses in age, sex, socioeconomic status, perceived neighborhood disorder, moderate-to-vigorous physical activity, or BMI, so no imputation was applied.

Table 1 presents means of the full perceived neighborhood disorder scale, as well as physical disorder, social disorder and prosocial environment subscales for the full sample. On average, the participants averaged in the middle-range of values of perceived neighborhood disorder and the various subscales.

**Table 1:** Descriptives of the perceived neighborhood disorder scale and subscales

	**Full sample (N = 101)**
^**±**^**Neighborhood disorder (15 – 60)**	26.81 ± 8.96
Physical disorder subscale (4 – 16)	6.67 ± 2.91
Social disorder subscale (6 – 24)	8.29 ± 3.55
*Prosocial environment subscale (20–5)	11.86 ± 4.40

^±^Neighborhood disorder and subscales, values in parentheses represent range of possible values.

*Prosocial environment is reverse coded, with lower scores representing a more prosocial environment.

Table 2 presents the characteristics of the participants for the full sample and by quartile of neighborhood disorder, where higher quartiles indicate greater perceived neighborhood disorder. There were no significant differences in the age or sex of participants by quartile of disorder. A significantly greater percentage of overweight and obese children were in the highest quartile of perceived neighborhood disorder. There were no significant differences in total minutes spent in physical activity at any activity level by quartile of perceived neighborhood disorder. There were also no significant differences in self-reported minutes spent participating in physical activities (i.e. basketball, football, dance, walking) by quartile of perceived neighborhood disorder. There were no significant differences in income by quartile of perceived neighborhood disorder. There were also no significant differences in education levels by perceived neighborhood disorder; and almost half of the adolescents in the sample resided in households where an adult had obtained a college degree or higher.

**Table 2:** Demographic characteristics, body mass index and physical activity by quartile of perceived disorder, higher quartiles indicate higher perceived disorder

	**Full sample**	**Quartile 1**	**Quartile 2**	**Quartile 3**	**Quartile 4**	**Test statistic (**^**a**^**Chi-square or **^**b**^**F-statistic) and p-value**
	**N = 101**	**Perceived disorder**	**Perceived disorder**	**Perceived disorder**	**Perceived disorder**	
		**n = 23**	**n = 26**	**n = 20**	**n = 32**	
**Age**	13.99 ± 1.31	13.73 ± 1.28	14.07 ± 1.38	14.05 ± 1.27	14.06 ± 1.34	0.36 ^a^, 0.784
**Sex** (% female)	55	47.83	61.54	65.00	46.88	2.57 ^a^, 0.462
**Income**						8.94 ^a^, 0.177
≤ $30,000	24 (23.76)	5 (21.74)	3 (11.54)	5 (25.00)	11 (34.38)	
$30,001 - $60,000	45 (44.55)	8 (34.78)	14 (53.85)	7 (35.00)	16 (50.00)	
$60,001+	32 (31.68)	10 (43.48)	9 (34.62)	8 (40.00)	5 (15.63)	
**Highest education level**						0.331 ^a^, 0.565
**Attained in the household (by any adult)**						
< High school	4 (3.96)	2 (8.70)	1 (3.85)	0	1 (3.13)	
High school	9 (8.91)	1 (4.35)	2 (7.69)	2 (10.00)	4 (12.50)	
Some college	39 (38.61)	8 (34.78)	6 (23.08)	13 (65.00)	12 (37.50)	
College+	49 (48.51)	12 (52.17)	17 (65.38)	5 (25.00)	15 (46.88)	
**Weight** (kg)	65.30 ± 19.77	59.12 ± 13.44	63.37 ± 16.26	61.92 ± 9.71	73.42 ± 27.47	
**Height** (inches)	64.77 ± 3.71	64.05 ± 4.04	65.29 ± 3.95	64.61 ± 2.83	64.97 ±3.81	
**Obesity status** n (%)						5.77 ^a^, 0.016
Normal Weight	57 (56.44)	14 (60.87)	16 (61.54)	23 (60.00)	15 (46.88)	
Overweight	26 (25.74)	8 (34.78)	7 (26.92)	7 (35.0)	4 (12.50)	
Obese	18 (17.82)	1 (4.35)	3 (11.54)	1 (5.0)	13 (40.63)	
**Physical activity **(total minutes)						
Sedentary	1050.34 ± 84.52	1040 ± 87.82	1045.57 ± 92.91	1076.43 ± 71.52	1046.38 ± 82.75	0.71 ^b^, 0.546
Light	356.28 ± 75.25	356.66 ± 77.58	368.09 ± 84.34	329.80 ± 51.60	358.20 ± 76.86	0.96 ^b^, 0.417
Moderate	26.90 ± 18.46	32.19 ± 20.78	24.06 ±17.50	19.92 ± 14.10	29.33 ± 18.76	5.20 ^b^, 0.157
Vigorous	2.81 ± 4.05	4.00 ± 5.02	2.26 ± 3.24	1.96 ± 2.39	2.87 ± 4.55	3.19^b^, 0.362
**Self-reported physical activity**^**d**^ (total minutes)						
Basketball	44.83 ± 66.11	62.54 ± 87.59	43.66 ± 51.42	26.02 ± 59.93	38.37 ± 55.09	0.63^b^, 0.600
Dance	8.08 ± 20.20	6.43 ± 21.57	0	19.90 ± 29.30	8.47 ± 18.96	1.28^b^, 0 .290
Football	12.04 ± 28.96	22.23 ± 42.75	3.75 ± 10.60	19.89 ± 35.22	5.16 ± 14.25	1.51^b^, 0.224
Walking	11.03 ± 18.31	13.92 ± 25.17	14.20 ± 17.63	0	11.29 ± 14.9	1.07^b^, 0.372

Obesity Status categories were developed using the International Obesity Taskforce estimates that correspond to overweight (25 kg/m^2^) and obesity (30 kg/m^2^) at 18 years of age.

^a, b ^refer to the test statistic used to test for significant differences between quartiles.

^d^Self-reported physical activity logs were completed by the adolescents, the data reported represent the average minutes spent in the most frequently reported activities for boys and girls.

### Mediation model

**Figure 1:**
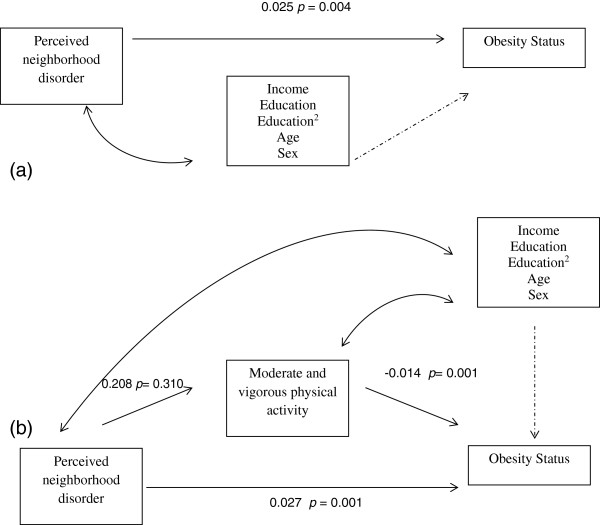
**Simple mediation model for the relationships between perceived neighborhood disorder and obesity status. **Panel (**a**) path estimates for the direct effect of perceived neighborhood disorder on obesity status (controlling for income, education, education^2 ^-to control for the non-linear effects of education, age and sex) (**b**) path estimates of the indirect effect of perceived neighborhood disorder on obesity status (controlling for income, education, education^2 ^-to control for the non-linear effects of education, age and sex).

Figure 1, Panel a presents the unstandardized beta coefficients for the unmediated association of perceived neighborhood disorder with obesity status, while controlling for income, education, age and sex. Perceived neighborhood disorder was significant and positively related to obesity status. Panel b of Figure 1 presents the unstandardized beta coefficients for the model examining whether moderate-to-vigorous physical activity mediated the relationship of perceived neighborhood disorder on obesity status. Perceived neighborhood disorder was not significantly related to moderate-to-vigorous physical activity. However, moderate-to-vigorous physical activity was significant and inversely associated with obesity status. The direct path of perceived neighborhood disorder remained statistically significant and there was no evidence that moderate-to-vigorous physical activity mediated the relationship of perceived neighborhood disorder on obesity status (Table 3). We also examined whether vigorous physical activity mediated the associations of perceived neighborhood disorder on obesity status (data not shown) and found results similar to those presented.

**Table 3:** Indirect effects of perceived neighborhood disorder on obesity status among adolescents (unstandardized)

	**Bias-corrected point estimate (ab)**	**S.E.**	**Bias-corrected accelerated bootstrap 95% confidence interval**	
*Model summary for Obesity Status*			Lower	Upper
Moderate & vigorous physical activity	0. 000	0.003	-0.010	0.004

## Discussion

This study set out to examine if subjective reports of perceived neighborhood disorder were associated with objectively measured physical activity and obesity status among African American adolescents. For the current study, accelerometer data indicated that the adolescents were engaged in low levels of moderate-to-vigorous physical activity (less than 30 minutes daily) and almost half of the adolescents (43 percent) were either overweight or obese. Using the conceptual model developed by Burdette and Hill [32], it was hypothesized that living in disordered neighborhoods would affect obesity status through less participation in physical activity. The current research provides partial support for the conceptual model and suggests that while adolescents’ perceptions of neighborhood disorder are significantly related to obesity status, physical activity may not be a significant mediator of this relationship.

The current study findings are supported by the literature evaluating relationships of neighborhood social context on obesity. Evenson and colleagues assessed the relationship between neighborhood factors and BMI for adolescent girls and their findings suggest that neighborhood factors such as low levels of crime, seeing other children playing outside, and the availability of recreational facilities are associated with lower BMI [33]. Although they note that the influence of neighborhood factors is minimal, the neighborhood context may be important and may operate to protect adolescent girls from obesity [33]. In the current study, the associations are also minimal, but statistically significant. While some research studies do not find significant associations of neighborhood context with obesity among children [29,44], the associations observed during adolescence may suggest that the obesogenic effects of neighborhood disorder emerge over time. For the current study, adolescents who live in disordered neighborhoods are more obese and because obesity tracks into adulthood, they may have increased risks for adverse health outcomes later in life. Future studies should longitudinally assess perceived neighborhood disorder characteristics and childhood adiposity to examine the timing and extent to which perceived neighborhood disorder characteristics begin to develop into increased obesity risk among youth.

Contrary to the expected findings outlined by the conceptual model, we did not find significant associations between perceived neighborhood disorder and objectively measured physical activity. While a few studies report significant associations between neighborhood disorder and physical activity, these studies typically rely upon self-reported physical activity assessments [32,45-47]. However, the use of accelerometry in the current study provides an objective measurement of physical activity and contributes to the literature that suggests a null relationship. Previous studies using objectively measured physical activity also indicate that neighborhood safety and disorder may not be associated with child and adolescent physical activity levels [48]. Studies that examine area-level SES also do not find significant relationships with physical activity [24]. Cumulatively, these research findings may suggest that neighborhood social contexts are not associated with physical activity when physical activity is measured objectively.

However, there may be specific neighborhood features of social and physical disorder that directly affect physical activity. When we examined bivariate correlations between individual items from the perceived neighborhood disorder scale and physical activity (data not shown), specific elements of neighborhood disorder such as drug use in the neighborhood, was significantly and negatively associated with participation in moderate-to-vigorous physical activity among adolescents. Evenson and colleagues also examined specific social and built environment factors related to physical activity and showed that while perceived neighborhood crime is not associated with physical activity, factors such as street lights, recreational facility access, and the presence of other children playing outside are associated with greater non-school related physical activity participation for adolescent girls [33]. Therefore, while perceived neighborhood social disorder such as crime levels may decrease feelings of safety, it appears that population density on the streets may provide a buffering effect [33]. Future studies that incorporate accelerometry should separate school based physical activity from non-school based physical activity and disaggregate features of neighborhood disorder to determine the extent to which neighborhood factors may affect physical activity among children. This type of research is especially important if children get most of their physical activity at school [26].

The current study findings provide support for the inverse association of moderate-to-vigorous physical activity with obesity status. Although most children in this study did not meet daily recommendations of at least 60 minutes of moderate-to-vigorous physical activity, their participation in physical activity appeared to be protective against obesity. We also examined other types of physical activity data from the accelerometers including models of participation in light or vigorous physical activity in association with obesity, but found no significant associations (data not shown). As a supplement to the objective accelerometer data, we also examined adolescents’ qualitative reports of the types of physical activities that they participated in and the duration of these activities. Among adolescent girls, the most frequently reported physical activities were walking for exercise, dance, and basketball. Among boys, the most frequently reported activities were basketball, football and walking for exercise. Although adolescents subjectively reported being physically active, the accelerometer data reflected that the activity levels were insufficient to meet the recommended physical activity guidelines. However, research findings from a nationally representative sample of children and adolescents, indicate that children and adolescents are more likely to engage in unstructured sporadic physical activity than structured physical activity [49]. These sporadic bouts of physical activity are inversely associated with overweight and obesity and are independent of total physical activity. This suggests it is important to measure both sporadic physical activity in addition to a measure of moderate-to-vigorous physical activity [49].

Because physical activity did not appear to mediate the association of perceived neighborhood disorder and obesity, future research should analyze the additional neighborhood disorder pathway of diet, which is a key component of the perceived neighborhood disorder conceptual model outlined by Burdette and Hill [32]. Fast food density and grocery store access, and fruit and vegetable availability are known to differ by neighborhood context [50-55] and these neighborhood features are associated with poorer diet quality and adolescent obesity [56,57]. An exploration of the relationship to diet in conjunction with the neighborhood pathway through physical activity may provide more evidence for the mechanisms through which perceived neighborhood disorder affects adolescent obesity.

While informative, this study is not without limitation. The sample size is relatively small and was obtained from a limited geographical region. As such, the current findings may not be generalizable to African American adolescents as a whole. Further, the sample data are cross-sectional in nature and therefore causality could not be established. Also, while the use of accelerometer data strengthened the findings, the data presented may not represent regular physical activity patterns of adolescents throughout the year. Additionally, research suggests that since children engage in very short bouts of sporadic vigorous physical activity, the 60 second epoch length of the accelerometery may underestimate the amount of moderate-to-vigorous physical activity patterns of the adolescents [58]. Further, this study did not include subjective or objective measures of stress as suggested by the conceptual model of Burdette and Hill [32]. Future work should incorporate these measures to examine the extent to which neighborhood disorder directly affects psychological and physiologic functioning.

Despite the study limitations, this research has significant strengths and contributes to the neighborhood and health literature by incorporating a conceptual model to examine the mechanisms through which perceived neighborhood disorder may affect obesity risk among African American adolescents, a population who experiences higher prevalence of obesity. Further, the inclusion of traditional measures of body mass index coupled with objective measures to assess physical activity, strengthen the current study findings and suggest that perceived neighborhood disorder and the low levels of physical activity observed among adolescents may contribute to obesity. Future intervention efforts to reduce obesity among African American adolescents should be developed to address strategies to increase physical activity and to modify features of the perceived neighborhood environment contexts that are directly associated with obesity.

## Conclusions

The current study findings highlight the associations of adolescents’ perceptions of neighborhood disorder and physical activity with obesity status among African American adolescents, a population who is at high risk for both low-physical activity participation and obesity. While the findings indicate that physical activity may not mediate associations of neighborhood disorder on body mass, both may play a role in contributing to obesity. Therefore, community-based interventions to decrease obesity among African American adolescents should devote attention to both neighborhood perceptions and physical activity outcomes.

## Competing interests

The authors declare that they have no competing interests.

## Authors’ contributions

AJK, HT and MLB were involved in the conception and design. AJK conducted the analysis. AJK and HT wrote the first version. OA was involved with interpretation of the physical activity data. All of the authors were involved with drafting and revising the article and final approval.

## Pre-publication history

The pre-publication history for this paper can be accessed here:

http://www.biomedcentral.com/1471-2458/13/440/prepub
